# Exploratory review on the effect of *Astragalus mongholicus* on signaling pathways

**DOI:** 10.3389/fphar.2024.1510307

**Published:** 2024-12-11

**Authors:** Inmaculada Xu Lou, Xinyi Yu, Qilan Chen

**Affiliations:** Department of Cardiology, Hangzhou Hospital of Traditional Chinese Medicine, Hangzhou, Zhejiang, China

**Keywords:** *Astragalus mongholicus*, signaling pathways, astragaloside IV, formononetin, polysaccharides

## Abstract

**Background:**

*Astragalus mongholicus* Bunge [Fabaceae; *Astragali radix*] (AM), a traditional Chinese medicinal (TCM) botanical drug, has been used for centuries and is gaining growing recognition in medical research for its therapeutic potential. The currently accepted scientific name is Astragalus mongholicus Bunge, with Astragalus membranaceus Fisch. ex Bunge recognized as a taxonomic synonym. This review explores the most relevant scientific studies on AM, focusing on its chemical composition, mechanisms of action, and associated health benefits.

**Main body:**

AM is commonly used in clinical practice to treat diabetes mellitus, cardiovascular diseases, oncological processes, lipid metabolism disorders, and ulcerative colitis. Recent research has investigated its potential as a product for anti-aging purposes. These therapeutic effects are attributed to the interactions of bioactive metabolites such as Astragaloside IV, Formononetin, and polysaccharides, with various signaling pathways, leading to the activation or inhibition of gene expression. This review aims to map the signaling pathways affected by these metabolites and their effects on different pathologies. Studies suggest that these metabolites act on signaling pathways such as TLR4/MyD88/NF-κB, PI3K/AKT, RNA expression, and tumor receptors. However, further research is necessary to validate the findings in human trials with better methodological quality.

**Conclusion:**

AM is rich in bioactive metabolites that interact with various signaling pathways, modulating diseases such as diabetes mellitus type 2, cardiovascular diseases, cancer, lipid metabolism disorders, and ulcerative colitis. Although promising, the majority of the studies are conducted *in vitro* and animal models, and more rigorous human trials are needed to determine the therapeutic potential of AM.

## Introduction

*Astragalus mongholicus* Bunge (AM), also known as “Huang Qi” in TCM, is a medicinal plant belonging to the legume family (Fabaceae). *Astragalus mongholicus* Bunge [Fabaceae; *Astragali radix*] is the species currently accepted according to modern taxonomy. Historically, it has been referred to as *Astragalus membranaceus* Fisch. ex Bunge in various publications. Native to Asia, particularly China and Mongolia, this plant has been valued for centuries for its immunomodulatory, antioxidant, and adaptogenic properties. The roots, which are the medicinally used part, contain active metabolites such as saponins, flavonoids, and polysaccharides. In traditional medicine, AM is used to strengthen the immune system, improve physical endurance, and promote longevity and vitality. It is also studied for its potential to reduce fatigue and protect organs from damage caused by oxidative stress (Tang and Huang, 2022; Li et al., 2022; Dong et al., 2023).

AM is often used in clinical practice to treat various pathologies and plays an important role in the international health trade. The composition of AM is quite complex, as it has more than 180 bioactive metabolites, including triterpenoids, saponins, sapogenins, flavonoids, glycosides, polysaccharides, phytosterols, fatty acids, etc. (Su et al., 2021; Li et al., 2018). These metabolites have anti-oxidation, anti-inflammation, immune regulatory, anti-cancer, lipid-lowering, hypoglycemic, anti-fatigue, antiviral, and anti-aging effects (Zhang et al., 2022). These effects are produced by the interaction between the bioactive metabolites of AM and different signaling pathways and the activation or inhibition of gene expression (Su et al., 2021; Jin et al., 2020; Gong et al., 2018; Sheng et al., 2021; Zhong et al., 2022).

Astragaloside IV (AS-IV) belongs to the group of saponins and is the main metabolite in AM (Su et al., 2021; Zheng et al., 2018). It has been shown to have numerous pharmacological properties, such as immunoregulation, antioxidant, anticancer, antiviral (Li et al., 2018), neuroprotection, liver protection, and improved sensitivity to cancer treatment. These effects are due to the interaction of AS-IV with several signaling pathways, including Raf-MEK-ERK, EGFR-Nrf2, Akt/PDE3B, AMPK, NF-κB, Nrf2, PI3K/Akt/mTOR, PKC-α- ERK1/2- NF-κB, IL-11/STAT3, Akt/GSK-3β/β-catenin, JNK/c-Jun/AP-1, PI3K/Akt/NF-κB, miRNA-34a/LDHA, Nox4/Smad2, JNK, and NF-κB/PPARγ (Zhang et al., 2020). Formononetin is a phytoestrogen that is increasingly being studied in the field of cancer, and *in vitro* studies have shown that it can prevent cancer progression by activating apoptosis, modulating the cell cycle, and modulating certain signaling pathways related to metastasis (Jiang et al., 2019). Other bioactive metabolites found in greater proportions are polysaccharides, which have been shown to have antioxidation, antihypertensive, antitumor, and immunomodulatory properties (Zhang R. et al., 2018).

AM can provide numerous health benefits; however, the exact mechanisms of action remains unknown (Shi et al., 2021). Therefore, the objective of this exploratory review was to map the signaling pathways in which AM bioactive metabolites act and their effects on different pathologies.

## Search method

**FIGURE 1:**
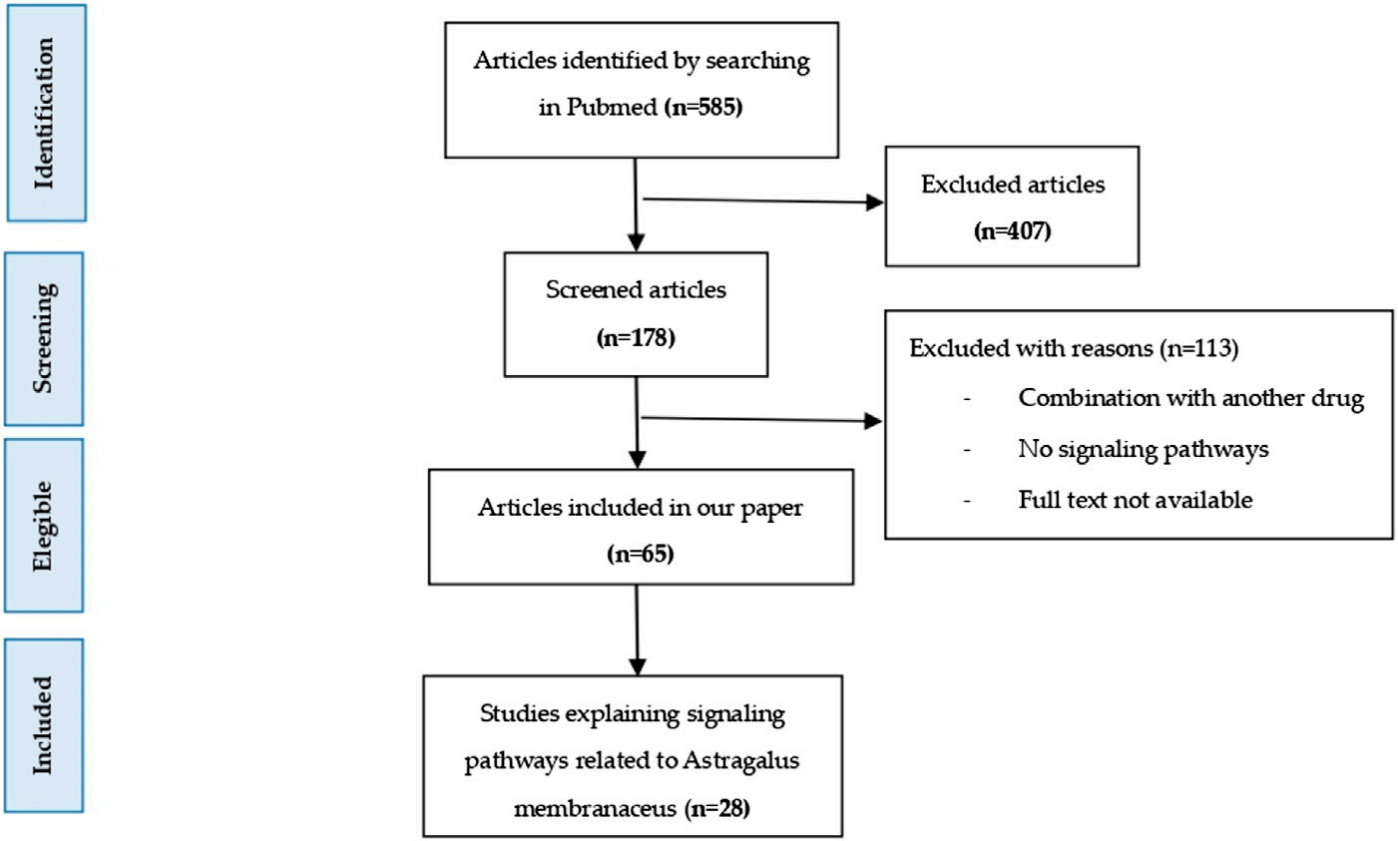
Flowchart.

**FIGURE 2:**
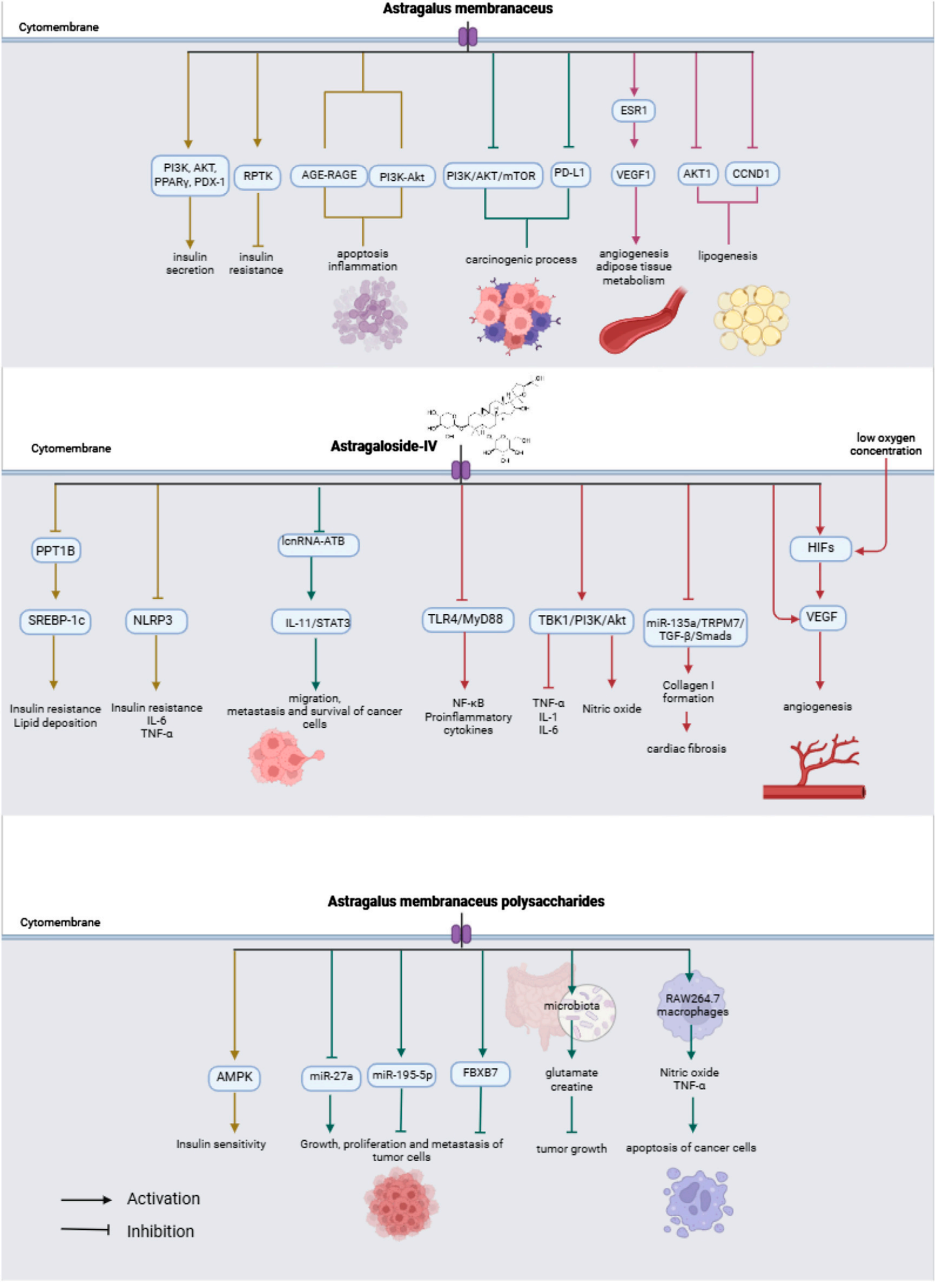
The mechanisms of AM and its main bioactive metabolites on health.

A bibliographic search of the PubMed database was performed. The keywords used were “Astragalus membranaceus”, “Huang qi”, “Astragaloside IV”, “Formononetin” and “signaling pathways”. In order for the results to be as up-to-date as possible, the search was filtered for studies published between September 2017 and October 2024, and published in English. 585 results were found. After performing the first filter by reading the title, those that did not have to do with the subject of the review (n = 407) were eliminated, of which 178 articles remained. After reading the abstract and full text, studies in which AM was administered in combination with another drug, studies that did not explain any signaling pathway, and articles whose full text could not be accessed were eliminated. In this bibliographic review, 65 studies were used, of which 28 explained the different signaling pathways in which the active metabolites of AM interact. A flowchart is shown in Figure 1. Table 1 summarize the data obtained in this study. Figure 2 shows a summary of the effects of AM on health.

**TABLE 1:** Collection of data from articles included in the review.

Disease	Metabolite	Method	Signaling pathway	Effect	References
Diabetes mellitus	AM	Network pharmacology	AGE-RAGE, PI3K-Akt	↓apoptosis, ↓inflammation, ↓monocyte adhesion	Jin et al. (2020)
	AM	Systematic review and meta-analysis		↓albuminuria, proteinuria, serum creatinine in diabetic kidney disease	Zhang et al. (2019a)
	AM	Network pharmacology	RPTK	↓insulin resistance	Li et al. (2019a)
	AS-IV	*In vitro*	PPT1B	↓insulin resistance, ↓lipid accumulation in HepG2 cells	Zhou et al. (2021)
	AS-IV	Mice AS-IV 15–30 mg/kg	NLRP3	↓blood glucose, ↑insulin, ↓ IL-6, ↓TNF-α	Zhang et al. (2019b)
	AMP	*In vitro*	AMPK	↑insulin sensitivity, ↑proliferation and differentiation of 3T3-L1 preadipocytes	Zhang et al. (2018a)
	AMP	Mice AMP 800 mg/kg intra-gastrically (purity 98%) Control Metformin		Enhanced control of body weight, blood glucose, and lipid levels, ↑liver function, ↑insulin sensitivity, and ↓levels of TNF-α, IL-6, IL-1β, and leptin	Sun et al. (2019)
	Isoflavonoids	*In vitro*	PI3K, AKT, PPARγ, PDX-1	↑insulin secretion in β-cells	Lee et al. (2019)
Myocardial ischemia	AS-IV	Systematic review and meta-analysis (animal models)		↑angiogenesis, ↑circulation, ↓oxidation, ↓inflammation, ↓apoptosis	Zheng et al. (2018)
Acute myocardial infarction	AS-IV	Rats AS-IV 80 mg/kg/day (purity 98%) Control captopril	TLR4/MyD88/NF-κB	↓inflammation, ↓TLR4, ↓MyD88, ↓NF-κB	Shi et al. (2021)
Vascular endothelial dysfunction	AS-IV	Rats AS-IV 40–80 mg/kg intragastric Control CMC-Na	TLR4/NF-kB	↑eNOS, ↑NO, ↓IL-6, ↓TNF-α	Leng et al. (2018)
Apoptosis of myocardial cells	AS-IV	*In vitro*	TLR4/NF-κB	↓NF-κB, ↓apoptosis of myocardial cells	Zhao et al. (2018)
Vasodilation	AS-IV	*In vitro*	PI3K/Akt/eNOS	↑vasodilation, ↑NO	Lin et al. (2018)
Myocardial ischemia	AS-IV	Review	MAPK, PI3K/AKT, Notch1, NF-κB	↓myocardial fibrosis, ↑myocardial contractility, ↑angiogenesis, ↓vascular endothelial dysfunction	Tan et al. (2020)
Cardiac hypertrophy	AS-IV	Mice AS-IV 10–20 mg/mg	TBK1/PI3K/AKT	Organ remodeling, ↓apoptosis, ↓inflammation, ↓TNF-α, ↓IL-1, ↓IL-6	Liu et al. (2018a)
Hypoxia	AS-IV	In vitro	VEGF	↑angiogenesis	Wang et al. (2021)
Cardiac fibrosis	AS-IV	Rats AS-IV 10 mg/kg/day	miR-135a-TRPM7-TGF-β/Smads	↓cardiac fibrosis, ↓collagen I	Wei et al. (2020)
Ischemic stroke	AS-IV	Rats AS-IV 40 mg/4 mL/kg/day	BNDF/TrkB	↑angiogenesis	Ni et al. (2020)
Myocardial ischemia	AMP	Rats AMP 250–750 mg/kg/day (purity 99.8%) *In vitro*		↓apoptosis, ↓ROS	Liu et al. (2018b)
Tumor and cancer	AM	Network pharmacology	CCL2, CXCL8, CXCL10, PTGS2	↑apoptosis of cancer cells	Chu et al. (2021)
	AM	*In vitro*	PD-LI, PD-I, AKT/mTOR/p70S6K	↑recognition of tumor cells by the immune system	Chang et al. (2020)
	AM	*In vitro* (cancer cells)	PI3K/AKT/mTOR	↑cytotoxicity, ↑apoptosis	Zhou et al. (2018)
	AS-IV	*In vitro*	lcnRNA-ATB, IL-11/STAT3	↓tumor cell migration	Li et al. (2018)
	AMP	Mice AMP 150–300 mg/kg Control 5-Fu		Modulation of T and B cells CD3^+^, CD4^+^, CD8^+^, promote immune system	Yu et al. (2021)
	AMP	Mice AMP 200 mg/kg/day		↓IL-10, ↓TGF-β, ↑CD8+ T cells	Ding et al. (2021)
	AMP	*In vitro* (cancer cells)	miR-195-5p	↓cell proliferation, ↓cell migration, ↓cell invasion	Tao et al. (2022)
	AMP	*In vitro* (cancer cells)	microRNA-27a/FBXW7	↑apoptosis, ↓cell proliferation	Guo et al. (2020)
	AMP	*In vitro* (cancer cells)		↑macrophage activation, ↑NO, ↑TNF-α, ↑apoptosis	Li et al. (2019b)
	Formononetin	Review		Apoptosis, ↓cell proliferation, ↓cell invasion	Tay et al. (2019)
	Formononetin	*In vitro* (cancer cells)	PI3K/AKT, STAT3	↓growth and invasion of cancer cells	Wang et al. (2018)
	Formononetin	*In vitro* (cancer cells)	Bax/Bcl-2	↓proliferation of cancer cells, ↓metastasis	Zhang et al. (2018b)
	Formononetin	Network pharmacology *In vitro*	ERK1/2	Enhance chemotherapy	Hu et al. (2023)
Hyperlipidemia	AM	Network pharmacology Mice AM 0.1 mL/10 g	AKT1, CCND1, VEGF1, ESR1	↓lipogenesis, ↑VEGF1	Wang et al. (2022)
	Formononetin	Mice	PPARγ, UCP1	↑thermogenesis	Nie et al. (2018)
	Flavones	Mice Flavones of AM 10–20 mg/kg/day Control High fat diet	miR-33	↓lipid dysregulation, ↓inflammation, ↓atherosclerosis, ↓NFκB	Ma et al. (2020)
Ulcerative colitis	AS-IV	Mice AS-IV 50–100 mg/kg/day	Th17/Treg cells	↓weight loss, ↓colon shortening, ↓ulceration, ↓inflammation, ↓IL-17A, ↓IL-21	Zhong et al. (2022)
Aging	AM	*Drosophila melanogaster* AM 1.25 mg/mL		↓ rate of telomere shortening	Zhang et al. (2022)
	AM	Review	Bcl-2	↓apoptosis of normal cells, ↑apoptosis of cancer cells	Liu et al. (2017)

Abbreviations. AM = astragalus mongholicus; AS-IV = Astragaloside IV, AMP = astragalus mongholicus polysaccharides.

### Reduction of insulin resistance and improvement of metabolism in type 2 diabetes

AM is often used in the treatment of diabetes mellitus type 2 (DM2) owing to its hypoglycemic effect. The metabolites found in AM can interact with the AGE-RAGE and PI3K-Akt signaling pathways, which are related to apoptosis, inflammation and monocyte adhesion. These signaling pathways modulate the evolution of complications during the development of DM2 (Jin et al., 2020), such as the deterioration of renal function. The use of AM in patients with diabetic kidney disease has been shown to decrease the levels of albuminuria, proteinuria and serum creatinine (Zhang L. et al., 2019). The antidiabetic effects of AM are highly associated with the activation of receptor protein tyrosine kinase (RPTK), however, the exact mechanism of action is unknown, since numerous genes are involved in this process. It has also been found to regulate lipid metabolism in DM2 and improve insulin resistance (Li J. et al., 2019).

Protein tyrosine phosphatase 1B (PPT1B) increases the transcriptional activity of SREBP-1c, resulting in lipid deposition in the liver, decreased glucose processing ability, and increased insulin resistance. On the other hand, AS-IV is an inhibitor of PPT1B, which helps to reverse this process. It regulates the metabolism of lipids, and carbohydrates and decreases insulin resistance, in a dose-dependent manner. In addition, according to X. Zhou et al., the lipid regulating effect of AS-IV is similar to that of atorvastatin, although further research is needed to corroborate this finding (Zhou et al., 2021). The development of gestational diabetes poses a risk to both the mother and fetus. This alteration in glucose metabolism strongly correlates with an abnormal inflammatory response. Zhang et al. induced diabetes in pregnant mice and subsequently administered AS-IV. A decrease in glucose levels and an increase in insulin levels were observed. Besides that, the expression of the NLRP3 inflammasome, which is related to insulin resistance, showed a clear decrease, and the expression of IL-6 and TNF-α was also reduced, in a dose-dependent manner (Zhang R. et al., 2019).

Abnormal metabolism in adipose tissue is known to be associated with the development of DM2. Zhang et al. found that *A. mongholicus* polysaccharides (AMP) can significantly increase insulin sensitivity by activating AMPK in 3T3-L1 adipocytes. This process involves the differentiation and proliferation of 3T3-L1 preadipocytes and the normalization of adipose tissue metabolism (Zhang R. et al., 2018). AMPs can also activate hepatic insulin signaling. Better control of body weight, and blood glucose and lipid levels, liver function, and insulin sensitivity was achieved. AMPs can decrease the levels of proinflammatory cytokines such as TNF-α, IL-6, IL-1β, and leptin. AMPs and metformin can activate insulin production through similar mechanistic pathways (Sun et al., 2019). In contrast, Lee et al. observed that AM isoflavonoids, including calycosin-7-O-β-d-glucoside, formononetin and adenosine, increased the expression of PI3K, AKT, PPARγ and PDX-1. This increased expression increases insulin secretion from pancreatic β-cells and lowers blood glucose (Lee et al., 2019).

### Improvement of cardiovascular health through Astragalus mongholicus

AM also has therapeutic effects on pathologies related to the cardiovascular system, including myocardial fibrosis, cardiac hypertrophy, viral myocarditis, cardiomyopathy, hypertension, atherosclerosis, and hypertensive nephropathy (Su et al., 2021; Xu et al., 2022; Zhang et al., 2023; Meng et al., 2023). AS-IV can significantly decrease the size of the myocardial infarction zone, promote angiogenesis, improve circulation, and exert antioxidant, anti-inflammatory, and anti-apoptotic effects (Zheng et al., 2018). TLR4/MyD88 modulates the immune response and activates the expression of NF-κB and proinflammatory cytokines related to acute myocardial infarction. AS-IV has the potential to avert instances of acute myocardial infarction through the suppression of the TLR4/MyD88/NF-κB signaling pathway, a pathway intricately linked to myocarditis and myocardial fibrosis. Its inhibition also decreases the production of collagen I, collagen II, collagen III and HYP (Shi et al., 2021). Apart from that, AS-IV can suppress the TLR4/NF-kB signaling pathway, improve vascular endothelial dysfunction due to hyperglycemia, increase eNOS expression and NO production, prevent myocardial cell apoptosis and reduce the expression of biomarkers of inflammation, such as IL-6, TNF-α, VCAM-1, ICAM-1, TLR4, and NF-kB p65 (Leng et al., 2018; Zhao et al., 2018).

AS-IV may also enhance vasodilation via the PI3K/Akt/eNOS signaling pathway. This signaling pathway increases nitric oxide production, leading to the relaxation and dilation of blood vessels (Lin et al., 2018). AS-IV provides protection against damage to ischemic myocardial cells, suppresses myocardial hypertrophy and fibrosis, enhances myocardial contractility, ameliorates diastolic dysfunction, mitigates vascular endothelial dysfunction, and fosters angiogenesis. The safeguarding impact of AS-IV against ischemia and hypoxia is associated with modulation of the MAPK, PI3K/AKT, Notch1, and NF-κB signaling pathways (Tan et al., 2020). Cardiac hypertrophy can lead to arrhythmia, heart failure, and death. AS-IV protects against cardiac hypertrophy, possibly through the TBK1/PI3K/AKT signaling pathway, which is responsible for organ remodeling, including the heart. In cardiac hypertrophy, AS-IV can attenuate apoptosis and decrease levels of inflammatory cytokines, such as TNF-α, IL-1, and IL-6 in a dose-dependent manner (Liu Z. H. et al., 2018).

Hypoxia-inducible factors (HIFs) are transcription factors activated by changes in oxygen concentration. These transcription factors, including HIF-1α, regulate genes related to adaptation to hypoxia such as vascular endothelial growth factor (VEGF). Under hypoxic conditions, AS-IV can promote angiogenesis by activating HIF-1α and VEGF (Wang et al., 2021). In contrast, the miR-135a-TRPM7-TGF-β/Smad signaling pathway is associated with cardiac fibrosis. AS-IV can inhibit miR-135a, thereby inhibiting the signaling cascade and Collagen I formation, resulting in improvement of the fibrotic state (Wei et al., 2020). It was also confirmed that AS-IV has protective effects in the brain in cases of ischemic stroke since it promotes angiogenesis by activating the BNDF/TrkB signaling pathway. At the same time, neurogenesis in the hippocampus, learning and memory are also promoted after an ischemic stroke event (Ni et al., 2020).

AMPs also exhibits cardioprotective activities. In mice with myocardial ischemia, administration of AMP attenuated the increase in myocardial cell volume and reduced apoptosis. *In vitro*, it was observed that there was less activity of caspase-3 and greater activity of Bcl-2, which translates into inhibition of apoptosis and reduced production of reactive oxygen species (Liu D. et al., 2018).

### Antitumoral activity and induction of apoptosis

The combined use of chemotherapy and TCM provides greater benefits than chemotherapy alone. The combination of AM and chemotherapy achieves a better modulation of the immune system, the patient feels better physically, the survival rate increases, the side effects of chemotherapy are reduced, such as nausea, mucositis, or leukopenia, and there is a lower rate of tumor growth and increased apoptosis of cancer cells (Sheik et al., 2021; Li et al., 2020).

AM promotes apoptosis in colorectal cancer cells by inhibiting the pro-cancer genes CCL2, CXCL8, CXCL10 and PTGS2. This effect could be related to the antioxidant and anti-inflammatory properties of AM (Chu et al., 2021). In addition, in the tumor process, a tolerance response of the immune system is produced towards the tumor cells due to the union of PD-LI and PD-I (Xiong et al., 2024). When the expression of PD-LI decreases, the immune response increases. AM can reduce the expression of PD-LI in the cell membranes of tumor cells. This mechanism of action is related to the AKT/mTOR/p70S6K signaling pathway. Chang et al. observed that AM did not induce apoptosis of tumor cells; however, it slowed the reduction of tumor mass growth (Chang et al., 2020). Activation of the PI3K/AKT/mTOR signaling pathway is related to the formation of the carcinogenic process. AM extract can inhibit the proliferation of breast cancer cells by inhibiting PI3K/AKT/mTOR through its biometabolites with a dose-dependent effect, inducing cytotoxicity and apoptosis in these cells, and suppressing their growth and proliferation (Zhou et al., 2018). On the other hand, lncRNA-ATB promotes the migration, metastasis, and survival of hepatocellular carcinoma cells by activating the IL-11/STAT3 signaling pathway. AS-IV inhibits carcinogenesis by suppressing lncRNAs. Thus, IL-11/STAT3 is inhibited and the proliferation of cancer cells is interrupted (Li et al., 2018).

AMPs regulate the levels of CD3^+^, CD4^+^, CD8^+^ T, and B cells in certain types of tumors. They can also activate cells of the immune system, promote anaerobic metabolism in the tumor area, and induce apoptosis in tumor cells (Yu et al., 2021). In addition, AMPs can modulate the growth of dysbiosis-associated melanomas by modulating gut microbiota. This process could occur because these AMPs stimulate the microbiota to produce glutamate and creatine, which are metabolites that inhibit tumor growth. In contrast, AMPs can inhibit the expression of inflammatory cytokines such as IL-10 and TGF-β and activate CD8^+^ T cells, which are natural killers of tumor cells (Ding et al., 2021). AMPs can also enhance the activity of miR-195-5p, which is a miRNA responsible for the inhibition of cell invasion and migration of tumor cells (Tao et al., 2022). Besides that, miR-27a is an oncogenic miRNA responsible for the growth and proliferation of cancer cells, whereas FBXW7 is a tumor cell suppressor gene. Therefore, miR-27a and FBXW7 were negatively correlated, as when the expression of miR-27a increased, the activity of FBXW7 decreased, and *vice versa*. AMPs exhibit the ability to impede the growth, invasion, and migration of cancer cells, inducing apoptosis via the microRNA-27a/FBXW7 signaling pathway in a dose-dependent manner (Guo et al., 2020). AMPs can also activate RAW264.7 macrophages, which are responsible for apoptosis of cancer cell apoptosis by releasing cytotoxins such as nitric oxide or TNF-α (Li W. et al., 2019).

Formononetin exerts anticancer effects by modulating numerous signaling pathways involved in apoptosis, cell cycle, suppression of cell proliferation, and cell invasion (Tay et al., 2019). Formononetin can inhibit the growth and invasion of colon carcinoma cells by inhibiting the PI3K/AKT and STAT3 signaling pathways (Wang et al., 2018). Other studies suggest that formononetin may inhibit the oncological process by suppressing ERK1/2 (Hu et al., 2023). Furthermore, in ovarian cancer cells, it promotes apoptosis by increasing Bax/Bcl-2 expression in a dose-dependent manner (Zhang J. et al., 2018).

### Reduction of lipogenesis and induction of thermogenesis

VEGF1 genes are responsible for angiogenesis, reducing inflammation and regulating the metabolism of adipose tissue. ESR1 genes activates the expression of VEGF1. On the contrary, CCND1 and AKT1 genes expression promotes the development of obesity. Therefore, VEGF1, ESR1, CCND1, and AKT1 are genes closely associated with lipid metabolism and obesity. AM extract can improve the lipid profile by inhibiting lipogenesis and activating β-oxidation and lipolysis. AM stimulates the expression of VEGF1 and ESR1, and inhibits AKT1 and CCND1 (Wang et al., 2022). Furthermore, the PPARγ/UCP1 signaling pathway is responsible for inducing thermogenesis in adipocytes, especially in brown adipose tissue. AM can activate this process by binding formononetin-PPARγ, which forms a heterodimer with RXR and activates the expression of UCP1. Nie et al. administered formononetin to mice and found that it can lower body weight, thus opening a new avenue for obesity research (Nie et al., 2018). Apart from that, the flavones present in AM can reduce the size of atherosclerotic plaques and improve hepatic steatosis by modulating the lipid profile, inflammation, and monocyte adhesion. Flavones have the capacity to reduce NF-κB activity, lower levels of proinflammatory cytokines, and elevate levels of anti-inflammatory cytokines. A decrease in the expression of miR-33, a post-transcriptional regulator inflammation-related genes has been reported (Ma et al., 2020).

### Reduction of inflammation levels in ulcerative colitis

Ulcerative colitis is a disease characterized by excessive inflammation. According to scientific literature, Th17 cells are responsible for activating the inflammatory response, while Treg cells inhibit it. AS-IV may be a possible treatment pathway for this disease by modulating Th17/Treg cells, which are also related to immunosuppression and immune tolerance. Zhong et al., who used mice with ulcerative colitis, observed that in the group administered AS-IV, the response of Th17 cells was inhibited and Treg cells were activated. Also, lower levels of oxidative stress, decreased IL-17A and IL-21 inflammatory cytokines, and increased IL-10 and TGF-β1 anti-inflammatory cytokines were observed. On the other hand, at the macroscopic level, improvement was observed in the typical symptoms of ulcerative colitis, such as less weight loss and fewer ulcerations (Zhong et al., 2022).

### New findings: Astragalus as an anti-aging agent

Although aging is a biological process, AM extract has been shown in fruit flies *(Drosophila melanogaster)* to have the ability to slow telomere shortening. Some hypotheses show that the bioactive metabolites of AM can eliminate free radicals due to their antioxidant action. A potential candidate could be formononetin, which can activate the formation of endogenous antioxidants and improve the stabilization of the cellular structure (Zhang et al., 2022). AM polysaccharides, flavonoids and saponins can increase telomerase activity, decrease inflammation levels, modulate the immune system, prevent cancer, lower blood glucose and lipid levels, have hepatoprotective functions and increase diuresis. Besides that, the expression of Bcl-2 is related to the selectivity of these bioactive metabolites when it comes to acting on target cells, reducing the apoptosis of normal cells and promoting the apoptosis of cancer cells. However, the exact mechanism remains unknown (Liu et al., 2017).

## Limitations and future research

Experimental studies involving the use of botanical drugs present inherent challenges due to their complex composition, which consists of a variety of bioactive metabolites whose biological activity is not directed towards a single molecular target. Additionally, the composition of these plants is influenced by multiple factors, such as the preparation method, the species used, the agronomic conditions during cultivation, extraction methods, among others (Heinrich et al., 2022). Furthermore, a major challenge in studies using TCM is the lack of standardized protocols, as these vary depending on practitioners and researchers, making the replication difficult. Additionally, since TCM is a holistic treatment approach, it is challenging to measure and quantify using conventional scientific methods. TCM is primarily practiced in Asian countries, with its presence being more limited in Western countries. As a result, studies using herbal formulas within this framework are scarce. One of the main challenges faced by TCM is the need to reach a consensus in order to make studies more standardized (Zhou et al., 2019). In our study, we have not found human studies that analyze molecular pathways associated with the use of AM. After filtering the articles, we identified very few studies addressing this topic. However, AM is a drug commonly used in clinical practice in several Asian countries, where its therapeutic efficacy has been reported. This situation reflects a significant gap in knowledge that should be explored, not only in relation to AM but also regarding other plants used in TCM (Chen et al., 2019; Zhang et al., 2017).

AM is a TCM botanical drug that contains a wide variety of bioactive metabolites that can interact with various signaling pathways and modulate diseases. However, because phytotherapy is a field of study that is still booming, there are still gaps in the knowledge that needed to be explored. Additionally, most of the research on phytotherapy is carried out *in vitro* and in animal models, while relatively few studies carried out in humans have a low methodological quality. Although the studies included in this review provide evidences about the usefulness of AM in the treatment of diseases, the signaling pathways in humans are more complex that in cell and animal models (Tan et al., 2020); therefore, there is still a long way to go. This review opens the doors to new ways to treat diseases that are currently highly prevalent; however, additional research is imperative to comprehensively elucidate the complete mechanism of AM effects on human biology (Guo et al., 2020). There is a recognized need for further studies in humans to gain a deeper understanding of the mechanisms of action of AM in this population.

## Conclusion

AM influences various molecular pathways associated with diabetes, cardiovascular diseases, oncological processes, among others. However, most of the available studies have been conducted in animal models or *in vitro*. Further research in humans is needed to gain a more precise understanding of the mechanism of action of AM in this population.
